# Deficiency of Peptidylglycine-alpha-amidating Monooxygenase, a Cause of Sarcopenic Diabetes Mellitus

**DOI:** 10.1210/clinem/dgae510

**Published:** 2024-08-13

**Authors:** Alice Giontella, Mikael Åkerlund, Kevin Bronton, Cristiano Fava, Luca A Lotta, Aris Baras, John D Overton, Marcus Jones, Andreas Bergmann, Paul Kaufmann, Yulia Ilina, Olle Melander

**Affiliations:** Department of Clinical Sciences Malmö, Lund University, 205 02 Malmö, Sweden; Department of Clinical Sciences Malmö, Lund University Diabetes Center, Lund University, 205 02 Malmö, Sweden; Department of Clinical Sciences Malmö, Lund University, 205 02 Malmö, Sweden; Department of Clinical Sciences Malmö, Lund University Diabetes Center, Lund University, 205 02 Malmö, Sweden; Clinical Research Center, Skåne University Hospital, 205 02 Malmö, Sweden; Department of Clinical Sciences Malmö, Lund University, 205 02 Malmö, Sweden; Department of Emergency and Internal Medicine, Skåne University Hospital, 205 02 Malmö, Sweden; Department of Clinical Sciences Malmö, Lund University, 205 02 Malmö, Sweden; Department of Medicine, University of Verona, 37134 Verona, Italy; Regeneron Genetics Center, Regeneron Pharmaceuticals, Tarrytown, NY 10591, USA; Regeneron Genetics Center, Regeneron Pharmaceuticals, Tarrytown, NY 10591, USA; Regeneron Genetics Center, Regeneron Pharmaceuticals, Tarrytown, NY 10591, USA; Regeneron Genetics Center, Regeneron Pharmaceuticals, Tarrytown, NY 10591, USA; PAM Theragnostics GmbH, 16761 Hennigsdorf, Germany; PAM Theragnostics GmbH, 16761 Hennigsdorf, Germany; PAM Theragnostics GmbH, 16761 Hennigsdorf, Germany; Department of Clinical Sciences Malmö, Lund University, 205 02 Malmö, Sweden; Department of Clinical Sciences Malmö, Lund University Diabetes Center, Lund University, 205 02 Malmö, Sweden; Clinical Research Center, Skåne University Hospital, 205 02 Malmö, Sweden; Department of Emergency and Internal Medicine, Skåne University Hospital, 205 02 Malmö, Sweden

**Keywords:** diabetes, sarcopenia, genetics, PAM, insulin secretion

## Abstract

**Context:**

Peptidylglycine-α-amidating monooxygenase (PAM) is a critical enzyme in the endocrine system responsible for activation, by amidation, of bioactive peptides.

**Objective:**

To define the clinical phenotype of carriers of genetic mutations associated with impaired PAM-amidating activity (PAM-AMA).

**Design:**

We used genetic and phenotypic data from cohort studies: the Malmö Diet and Cancer (MDC; 1991-1996; reexamination in 2002-2012), the Malmö Preventive Project (MPP; 2002-2006), and the UK Biobank (UKB; 2012).

**Setting:**

Exome-wide association analysis was used to identify loss-of-function (LoF) variants associated with reduced PAM-AMA and subsequently used for association with the outcomes.

**Patients or Other Participants:**

This study included n∼4500 participants from a subcohort of the MDC (MDC-Cardiovascular cohort), n∼4500 from MPP, and n∼300,000 from UKB.

**Main Outcome Measures:**

Endocrine-metabolic traits suggested by prior literature, muscle mass, muscle function, and sarcopenia.

**Results:**

Two LoF variants in the *PAM* gene, Ser539Trp (minor allele frequency: 0.7%) and Asp563Gly (5%), independently contributed to a decrease of 2.33 [95% confidence interval (CI): 2.52/2.15; *P* = 2.5E^−140^] and 0.98 (1.04/0.92; *P* = 1.12E^−225^) SD units of PAM-AMA, respectively. The cumulative effect of the LoF was associated with diabetes, reduced insulin secretion, and higher levels of GH and IGF-1. Moreover, carriers had reduced muscle mass and function, followed by a higher risk of sarcopenia. Indeed, the Ser539Trp mutation increased the risk of sarcopenia by 30% (odds ratio 1.31; 95% CI: 1.16/1.47; *P* = 9.8E^−06^), independently of age and diabetes.

**Conclusion:**

PAM-AMA genetic deficiency results in a prediabetic sarcopenic phenotype. Early identification of PAM LoF carriers would allow targeted exercise interventions and calls for novel therapies that restore enzymatic activity.

Peptidylglycine α-amidating monooxygenase (PAM) is the only known enzyme responsible for the C-terminal amidation of peptide hormones, an essential step for their biological activation (1). Starting from a precursor and progressing through a glycinated intermediate, the final form is achieved via a 2-step amidation reaction catalyzed by 2 PAM's subunits, peptidyglycine alpha-hydroxylating monooxygenase (PHM) and peptidyl-alpha-hydroxyglycine alpha-amidating lyase (PAL) (2). Apart from the catalytic role exerted by the 2 subunits, PAM is implicated in the formation and stability of secretory granules of the endocytic pathway. This is evident in various cellular mechanisms in different tissues, such as insulin packaging and release from β-cells’ granules (3). as well as in the formation and stability of atrial and ventricular secretory granules, which store atrial and brain natriuretic peptides (4). It was shown that knockout mice for the *PAM* gene in atrial cardiomyocytes exhibit impaired natriuretic peptide secretion, despite that neither atrial natriuretic peptides nor brain natriuretic peptide require amidation, unlike many neuroendocrine peptides (4). However, the potential impact of PAM deficiency on natriuretic peptide secretion, as observed in animal studies, remains unexplored in humans.

Previous human studies have identified predicted nonsynonymous variants in the *PAM* gene associated with reduced insulin secretion and the risk of type 2 diabetes (T2D) (3, 5).

In addition, a reduced level of PAM-amidating activity (PAM-AMA) in plasma was observed in subjects with diabetes (1, 5-7). Furthermore, recent findings indicate that rare mutations of *PAM* are more prevalent in subjects experiencing pituitary gland hypersecretion, suggesting it may cause familial gigantism, ie, as characterized by GH excess (8).

In summary, prior literature has indicated the involvement of PAM deficiency in impaired insulin secretion and diabetes and potentially in excess GH secretion.

The aim of our study was to understand at the population level the effect of deleterious protein-coding variants affecting PAM enzyme functionality on endocrine metabolic phenotypes, including diabetes, insulin, and incretins levels before and after an oral glucose tolerance test (OGTT), hormones, and anthropometric traits, integrating data from 3 cohorts: the Malmö Diet and Cancer (MDC; n = 29 094), the Malmö Preventive Project (MPP; n = 4769) and the UK Biobank (UKB; n = 331 215).

## Research Design and Methods

**Figure 1.**
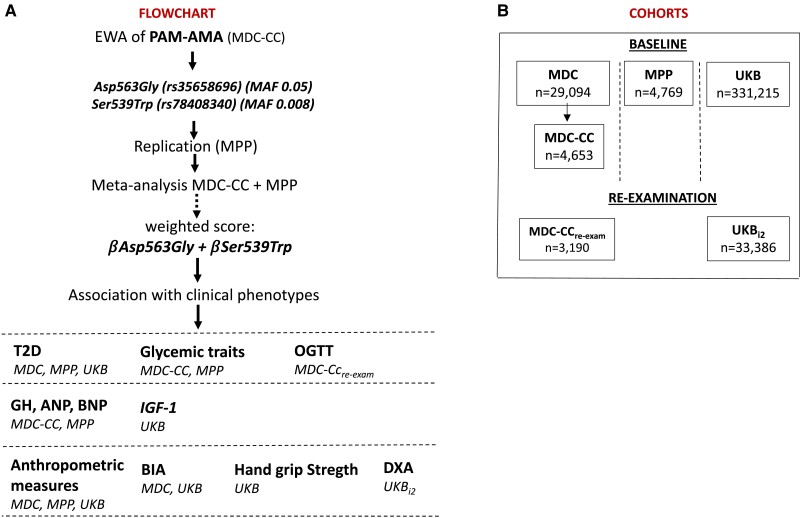
Overview of the cohorts and flowchart of the study design. Panel A shows an overview of the cohorts included in the analyses and their sample size. Panel B represents the flow chart of the study.

Abbreviations: ANP, atrial natriuretic peptide; BIA, bioelectrical impedance; BNP, brain natriuretic peptide; DXA, dual X-ray absorptiometry; EWA, exome-wide analysis; OGTT, oral glucose tolerance test; T2D, type 2 diabetes mellitus.

A graphical representation of the flowchart and the cohorts is presented in Fig 1A.

### Cohorts

The MDC is a prospective urban-based cohort study in which adults (born between 1923 and 1950) residing in Malmö (Sweden) were recruited between 1991 and 1996, for a total of 30 446 individuals participating in the baseline examination (9). The study protocol was approved by the Ethical Committee at the Medical Faculty at Lund University (approval number: LU 51/90); 6,103 MDC participants were randomly selected to take part in additional examinations aimed at studying the epidemiology of coronary artery atherosclerosis, known as the MDC-Cardiovascular cohort (MDC-CC) (10). Between 2007 and 2012, a reexamination of the MDC-CC was carried out involving ∼3700 individuals. The latter cohort is referred to in the text as the MDC-CC_re-exam_.

The MPP is a prospective cohort study started in 1974 as a screening of the Malmö adult population for cardiovascular risk prevention (11). A total of 33 346 individuals were recruited for the baseline examination, of which 18 240 underwent reexamination during 2002 and 2006 (ethics approval by the Region Board of Ethics at Lund No. 2009/633). We here used data from the reexamination.

The UKB is a prospective cohort study whose baseline examination started in 2009 and included ∼500 000 individuals (40-69 years) from the UK (12). Participants’ individual data were accessed with an approved application ID of 76 658 in compliance with the UKB Ethics Advisory Committee (https://www.ukbiobank.ac.uk/ethics/) and with the Swedish Ethical Review Authority (approval number: 2022-01085-01). Health records, self-reported records, International Classification of Diseases codes for disease diagnoses, drug use, blood biomarkers, bioimpedance, and imaging data were obtained during the assessment visits or through self-report surveys.

Informed consent was obtained from all the participants.

### Association of Coding Variants With PAM-AMA

PAM-AMA was assessed by a 2-step assay in the MPP, whose data and details were previously reported (1), and in the MDC-CC. Briefly, this assay uses a naturally occurring substrate of the PAM enzyme, the C-terminally glycine extended adrenomedullin to estimate PAM-AMA by detecting the substrate transformation in the product, the biologically active amidated adrenomedullin (1). PAM-AMA is expressed in units, where 1 AMA unit is defined as 1 µg adrenomedullin generated in 1L of sample in 1 hour.

The association of coding and predicted missense/loss-of-function (LoF) variants with PAM-AMA was explored in 4653 MDC-CC individuals with PAM-AMA measurement and genetics data. Detailed methods related to exome and genome sequencing and quality control steps are provided in the Supplementary Material (13). A linear regression model adjusted for age, sex, and the first 10 genetic principal components was used against the log-transformed and standardized PAM-AMA using *REGENIE* (v 3.1.1) (14). The significance threshold was set to the genome-wide association studies significance level of *P* < 5 × 10E^−8^. Variants with a minimum allele count of less than 5 were not included in the analysis. Among all significant signals resulting from the exome association analysis, the independent ones were identified by conditional analysis [see Supplementary Material (13)]. The associations of identified independent signals with PAM-AMA were then replicated in 4769 participants of the MPP cohort using the same statistical model. The estimates from the 2 cohorts were meta-analyzed using inverse variance weighting, and the beta coefficients from the affected/minor allele of selected variants were used as weights to compute a genetic score that was associated with the outcomes.

### Diabetes

T2D in the MDC and MPP was defined as having a fasting whole-blood glucose ≥6.1 mmol/L (corresponding to plasma glucose ≥7.0 mmol/L), self-reported or healthcare diagnosis (code E11 of the 10th version of the International Classification of Diseases), or use of diabetes medication (15). In the UKB, the criteria of having hemoglobin A1c (HbA_1C_) greater than 6.5% was also included.

### OGTT

All MDC-CC_re-exam_ participants underwent a OGTT in which blood samples were collected at fasting and 2 hours after intake of 75 g of glucose.

Plasma glucose, serum insulin, plasma glucagon, serum gastric inhibitory peptide (GIP), and plasma glucagon-like peptide-1 (GLP-1) were analyzed from blood samples between 0 and 120 minutes. The Hemocue Glucose System (HemoCue AB, Ängelholm, Sweden) was used to analyze plasma glucose and the Dako ELISA kit (Glostrup, Denmark) to analyze serum insulin. Plasma glucagon was assayed with RIA GL-32 K (Merck Millipore, Dermstadt, Germany) and serum GIP with the Millipore Human GIP Total ELISA #EZHGIP-54 K. Plasma GLP-1 (intact GLP-1 and the metabolite GLP-1 9-36 amide) were determined radioimmunologically, using an N-terminally specific guinea pig anti-GLP-1 antiserum (Linco Research, St Charles, MO, USA) for intact GLP-1 and a C-terminally directed antiserum (code no. 89390) for total GLP-1 (16).

### GH, IGF-1, Midregional Atrial Natriuretic Peptide, N-Terminal Brain Natriuretic Peptide

GH levels were measured in stored fasting plasma samples, frozen immediately at −80 °C during MDC-CC baseline examination, using a high-sensitivity chemiluminescence sandwich immunoassay (SphingoTec GmbH, Hennigsdorf, Germany), whose details were previously described (17). IGF-1 was measured in a serum sample collected for 431 718 participants of the baseline examination of the UKB.

Midregional atrial natriuretic peptide (MR-ANP) was measured using immunoluminometric sandwich assays targeted against amino acids in the midregion of the peptide (BRAHMS, Henningsdorf, Germany). N-terminal brain natriuretic peptide (Nt-BNP) was measured using the automated Dimension Vista Intelligent Lab System method (Siemens Diagnostics, Nürnberg, Germany) (18).

### Anthropometric Traits

Body mass index (BMI) was calculated by dividing weight (kg) by the square of height (m). Body fat percentage; body fat-free mass; and predicted muscle mass for right arm, left arm, right leg, left leg, and trunk were assessed in 492 952 individuals in the UKB using bioelectrical impedance (BIA; Tanita BC418MA).

Total body mineral content and density were assessed by dual-energy X-ray absorptiometry (iDXA instrument GE-Lunar, Madison, WI, USA) in 48 192 participants who underwent the imaging study started in 2014 (19).

Isometric grip strength for the right and left hand was measured in 499 206 participants with a Jamar J00105 hydraulic hand dynamometer.

### Sarcopenia

In this work, sarcopenia was defined as being in the bottom quintile of *all* the following 5 parameters: right-hand grip strength, left-hand grip strength, lean mass of arms (mean of right and left arm), legs (mean of right and left leg), and trunk estimated by BIA. Based on these criteria, the proxy variable for sarcopenia was created. For each measurement, the cut-offs for the quintiles were computed separately for women and men.

### Statistics

Individuals with genotypes whose data passed the quality control and had data on the outcome variables were included in the analyses, comprising specifically 29 094 from MDC; 4653 from MDC-CC; 3190 from MDC-CC_re-exam_; 4769 from MPP; 331 215 from UKB; and 33 386 from UKB_i2_ (Fig 1B).

The association of the PAM LoF score with outcomes was evaluated with linear/logistic regression for continuous/binary outcomes, respectively. Analyses were performed using R (v4.1.2; R Core Team 2021) and SPSS (version 29). Forestplots were created using R package *forestploter* (20)

## Results

The characteristics of the 3 cohorts are described in Supplementary Table S1 (13).

### Identification of Coding Variants Associated With PAM-AMA

We first aimed to identify coding and predicted missense/LoF variants independently associated with PAM-AMA.

**Figure 2.**
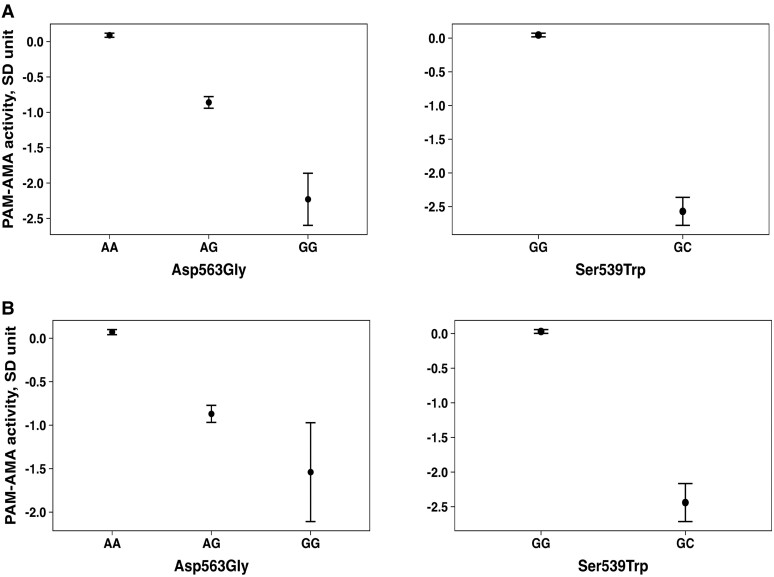
Difference in PAM-AMA activity across Asp563Gly and Ser539Trp genotypes. Difference in PAM-AMA activity across Asp563Gly and Ser539Trp genotypes in the MDC-CC cohort (A) and in the MPP cohort (B).

Abbreviations: MDC, Malmö Diet and Cancer cohort; MPP, Malmö Preventive Project cohort; PAM-AMA, peptidylglycine-α-amidating monooxygenase amidating activity.

Exome-wide analysis identified 2 LoFs, Ser539Trp (rs78408340, 5:103003035 C > G, GRCh38) and Asp563Gly (rs35658696, 5:103003107/A > G, GRCh38), both robustly associated with PAM-AMA, independently of each other [Supplementary Table S2, Supplementary Fig. S1, S2 (13)]. The effect alleles responsible for the 2 substitutions had a minor allele frequency (MAF) of 0.008 and 0.05, respectively, consistent among the 3 cohorts. The carriers of the LoF mutations showed a decrease per SD of log-transformed [95% confidence interval (CI)] PAM-AMA of (−2.64 [(−2.87)-(−2.41)]; *P* = 3.8E^−115^) for Ser539Trp, and (−0.98 [(−1.01)-(−0.89); *P* = 9.10E^−94^] for Asp563Gly in the MDC-CC; and (−1.83 [(−2.12)-(−1.53)]; *P* = 1.9E^−33^) SD for Ser539Trp, and (−0.82 [(−1.04)-(−0.92)]; *P* = 1.1E^−225^) for Asp563Gly in the MPP (Fig. 2A and 2B). The effect estimates for the meta-analysis of the 2 cohorts are shown in Table 1. To evaluate the combined impact of both variants, we computed a summed score based on the number of LoF effect alleles (0, 1, or 2) weighted for the meta-analyzed effect sizes on PAM-AMA for the 2 variants (Ser539Trp*2.33+ Asp563Gly*0.98), presented in Table 1.

**Table 1.** Association of the 2 LoF variants with PAM-AMA

	beta	95% CI	*P*-value
Ser539Trp	−2.33	−2.52, −2.15	2.50E-140
Asp563Gly	−0.98	−1.04, −0.92	1.12E-225

The table shows the estimates resulting from the meta-analysis of the associations of LoF variants with PAM-AMA in the MDC-CC and in the MPP cohort. The results for the linear regression are adjusted for age, sex, and the first 10 genetic principal components. PAM-AMA is expressed in standardized units.

Abbreviations: CI, confidence interval; LoF, loss-of-function; MDC-CC, MDC-CC: Malmö Diet and Cancer-Cardiovascular cohort; MPP, Malmö Preventive Project cohort; PAM-AMA, peptidylglycine-α-amidating monooxygenase amidating activity.

### Associations With Glucometabolic Traits

**Figure 3.**
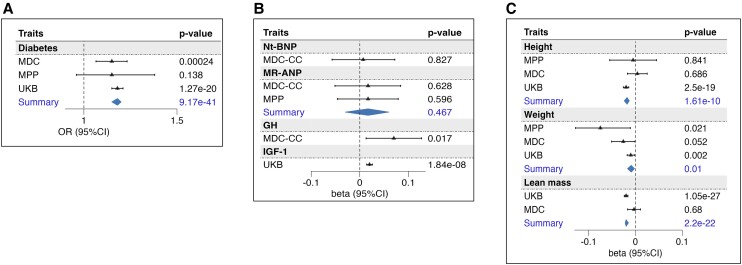
Association of PAM score with the outcomes. The forest plots represent the association of the PAM score with diabetes (A); natriuretic peptides, GH, and IGF-1 (B); GH-related anthropometric traits: height, weight, and lean mass (C). Betas represent the estimates of the linear association of each unit-increase of the score with respect to the standardized outcome. Odds ratios obtained through logistic regression refer to the risk associated with each unit of the PAM score. Summary refers to the estimates obtained from the meta-analysis of more cohorts. Abbreviations: MDC, Malmö Diet and Cancer cohort; MPP, Malmö Preventive Project cohort; MR-ANP, midregional atrial natriuretic peptide; Nt-BNP, N-terminal brain natriuretic peptide; PAM, peptidylglycine-α; UKB, UK Biobank.

Carriers with a higher PAM LoF score (expressed per unit increase) had higher fasting glucose concentration [per SD (95% CI), [0.017 (0.009-0.02); *P* = 1.08E^−05^] and higher HbA_1C_ [0.014 (0.012–0.017); *P* = 1.82E^−24^], as presented in Table 2. Each unit increase of the score corresponded to an odds ratio (OR) (95% CI) of 1.18 (1.15-1.20; *P* = 9.17E^−41^) for risk of T2D (Fig. 3A and Table 2).

**Table 2.** Association of the score with the outcomes

Outcome	beta/OR	95% CI		*P*-value
Diabetes				
UKB (cases 31995, controls 299220)	1.180	1.150	1.210	**2.60E-36**
MDC (cases 5796, controls 22376)	1.150	1.065	1.232	**2.40E-04**
MPP (cases 966, controls 4135)	1.150	0.960	1.380	.138
Summary	1.176	1.149	1.204	**9.17E-41**
HbA_1C_				
UKB	0.030	0.022	0.038	**2.92E-15**
MDC-CC	0.082	0.019	0.145	**.012**
Summary	0.014	0.012	0.017	**1.82E-24**
Glucose				
UKB	0.016	0.008	0.024	**2.72E-05**
MDC-CC	0.076	0.015	0.137	**.017**
MPP	0.009	−0.019	0.036	.535
Summary	0.017	0.009	0.020	**1.08E-05**
OGTT test				
Insulin 0	−0.030	−0.106	0.046	4.39E-01
Insulin 30	−0.157	−0.248	−0.066	**7.00E-04**
Insulin 120	−0.050	−0.135	0.036	2.54E-01
Delta ins30-ins0	−0.177	−0.272	−0.082	**2.00E-04**
Glucagon 0	0.016	−0.064	0.096	6.93E-01
Glucagon 120	0.026	−0.065	0.114	5.96E-01
GLP-1 0	−0.085	−0.170	0.001	**4.90E-02**
GLP-1 120	−0.067	−0.152	0.018	1.24E-01
SGIP 0	−0.057	−0.143	0.029	1.90E-01
SGIP 120	−0.069	−0.158	0.020	1.27E-01
NtBNP	0.007	−0.057	0.072	.827
MR-ANP				
MPP	0.017	−0.051	0.084	.628
MDC-CC	0.017	−0.046	0.079	.596
Summary	0.017	−0.03	0.06	.467
GH	0.07	0.013	0.127	**.017**
IGF-1	0.02	0.014	0.026	**1.84E-08**
Height				
UKB	−0.02	−0.026	−0.014	**2.50E-19**
MDC	0.004	−0.017	0.025	.686
MPP	−0.005	−0.055	0.045	.841
Summary	−0.018	−0.024	−0.013	**1.61E-10**
Weight				
UKB	−0.010	−0.018	−0.002	**.002**
MDC	−0.026	−0.051	−0.001	.052
MPP	−0.075	−0.128	−0.011	**.021**
Summary	−0.009	−0.017	−0.002	**.01**
BMI				
UKB	0.004	−0.004	0.029	.367
MDC	−0.035	−0.059	−0.011	**.021**
MPP	−0.088	−0.160	−0.017	**.015**
Summary	−0.012	−0.025	0.001	.07
WHR				
UKB	0.007	0.001	0.013	**.02**
MDC	−0.027	−0.052	−0.002	**.042**
MPP	−0.080	−0.134	−0.027	**.003**
Summary	0.004	−0.001	0.010	.126
BIA				
Body fat percentage				
UKB	0.011	0.007	0.015	**3.22E-12**
MDC	0.011	−0.003	0.025	.137
Summary	0.010	0.006	0.014	**1.60E-07**
Lean mass (kg)				
UKB	−0.020	−0.024	−0.016	**1.05E-27**
MDC	−0.003	−0.017	0.011	.68
Summary	−0.020	−0.022	−0.015	**2.20E-22**
Arm mass (mean right and left) kg	−0.019	−0.022	−0.016	**2.07E-32**
Leg mass (mean right and left) kg	−0.015	−0.017	−0.013	**7.10E-22**
Trunk lean mass kg	−0.020	−0.024	−0.016	**4.77E-33**
Trunk fat mass %	0.010	0.006	0.014	**1.40E-10**
DXA				
Total BMD g/cm2	−0.001	−0.018	0.016	.875
Total BMC g	−0.001	−0.014	0.013	**.0078**
VAT mass g	0.016	0.001	0.031	.03
Total fat mass g	0.004	−0.006	0.014	.42
Total fat free mass g	−0.030	−0.042	−0.018	**6.73E-07**
Legs total mass g	−0.010	−0.023	0.003	.008
Legs lean mass g	−0.050	−0.063	−0.037	**2.35E-09**
Legs fat mass g	0.001	−0.013	0.014	.896
Grip strength (muscle function)				
Hand grip strength (left) kg	−0.020	−0.026	−0.014	**2.48E-17**
Hand grip strength (right) kg	−0.020	−0.026	−0.014	**1.22E-17**

For those outcomes that were available in more than one cohort, the summary statistics of the meta-analysis were also reported. In bold values that are below the level of significance of *P* < .05 are indicated.

Abbreviations: BIA, bioelectrical impedance; BMC, body mineral composition; BMD, body mineral density; BMI, body mass index; CI, confidence interval; DXA, dual-energy X-ray absorptiometry; GIP, gastric inhibitory polypeptide; GLP-1: glucagon-like peptide-1; HbA_1c_, hemoglobin A1c; MDC, Malmö Diet and Cancer cohort; MDC-CC, Malmö Diet and Cancer-Cardiovascular cohort; MR-ANP, midregional atrial natriuretic peptide; Nt-BNP, N-terminal pro b-type natriuretic peptide; OGTT, oral glucose tolerance test; OR, odds ratio; UKB, UK biobank; WHR, waist-to-hip ratio; VAT, visceral adiposity tissue.

Individuals with a higher (per unit increase) PAM LoF score exhibited an insulin value 30 minutes after OGTT that was reduced [per SD (95% CI): −0.157 (−0.248/−0.066); *P* = .0007], along with a lower insulin incremental secretion (Table 2). A suggestive association was found with a per unit increase in PAM LoF score and decreased GLP-1 in the fasting state (−0.085 [−0.17/−0.001]; *P* = .049). No significant associations were found between PAM LoF score and either glucagon or GIP. Measures of glucagon and incretins at 30 minutes after the OGTT [Table 2 (13)] were not collected.

### Endocrine Disturbances and Related Phenotypes

As rare PAM LoF variants have previously been implied in familial gigantism (GH excess) and animal studies have shown that lack of atrial PAM expression results in the reduction of natriuretic peptide secretion (4, 8), we tested if the PAM LoF score was associated with GH/IFG-1 and natriuretic peptides level in plasma. While the PAM LoF score showed no relation with MR-ANP and Nt-BNP, it was significantly associated with both increased GH [0.07 (0.013–0.127); *P* = .017] and increased IGF-1 [0.02 (0.014–0.026); *P* = 1.8E^−08^] (Fig. 3B and Table 2).

Considering the findings indicating heightened activity in the GH/IGF-1 axis, we subsequently investigated if the PAM LoF score was associated with GH-related traits. Surprisingly, an association was found between PAM LoF score and both reduced height [per SD (95% CI): −0.018 [−0.024/−0.013)]; *P* = 1.61E^−10^], and reduced weight [−0.009 [−0.017/−0.002)]; *P* = .01], indicating an anthropometric phenotype paradoxically resembling GH deficiency rather than a phenotype resulting from high GH and IGF-1 (Fig. 3C).

Interestingly, in line with a GH-deficient phenotype, individuals with a higher PAM LoF score had reduced muscle mass and function. An increased PAM LoF score was associated with lower total lean mass (per SD (95% CI) (−0.020 [−0.022/−0.015]; *P* = 4.7E^−33^), lower arms lean mass (−0.020 [(−0.030/−0.014)]; *P* = 8.1E^−07^), lower legs lean mass (−0.015 [−0.017/−0.013]; *P* = 7.1E^−22^), and lower trunk muscle mass (−0.020 [−0.024/−0.016]; *P* = 4.8E^−33^), estimated through BIA. The association of PAM LoF score with decreased muscle mass was also confirmed by dual-energy X-ray absorptiometry measures (Table 2). Moreover, the PAM LoF score was associated with reduced muscle function assessed by the hand grip strength test for the left hand ((−0.02 [−0.03/−0.01)]; *P* = 2.5^−17^) and for the right hand (−0.02 [(−0.03/−0.01)]; *P* = 1.2E^−17^).

Finally, individuals having a lower PAM LoF score exhibited reduced body mineral composition, indicating a lower total bone mass. However, no significant differences were observed in terms of body mineral density [Table 2 (13)].

### Sarcopenia

**Figure 4.**
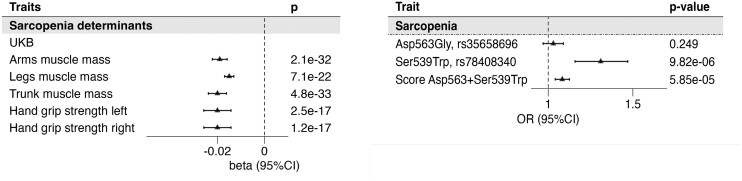
Association of the PAM score with traits used for sarcopenia definition and sarcopenia outcome. The forest plots represent the association of the PAM score with traits used to define Sarcopenia outcome in the UKB (A), which is then presented in (B). In panel B the association of the 2 variants and the score is presented. Betas represent the estimates of the linear association of each unit increase of the score with respect to the standardized outcome. Odds ratios obtained through logistic regression refer to the risk associated with each unit of the PAM score. Abbreviations: PAM, peptidylglycine-α; UKB, UK Biobank.

In the UKB cohort, 13 365 (4.0%) were classified as sarcopenic (according to the definition defined in the method) and were compared with of the nonsarcopenic population. Descriptive characteristics of the sarcopenic population are illustrated in Supplementary Table S3 (13). The PAM LoF score was associated with a higher risk of being sarcopenic with a per unit increment OR [95% CI] of 1.08 [1.04-1.13; *P* = 5.85E^−05^]. Of the 2 individual mutations, we found the Ser539Trp to be significantly associated with sarcopenia (1.31 [1.16-1.47]; *P* = 9.82E^−06^) but not the Asp563Gly (1.03 [0.97-1.09]; *P* = .25) [Fig. 4B and Supplementary Table S4 (13)].

### The Effect of Diabetes and age on the Association Between PAM LoFs and Sarcopenia

Given the robust association we found between PAM LoF with diabetes, we next investigated whether the effect on sarcopenia of PAM LoF mutations would act through diabetes, also motivated by the numerous potential mechanisms linking T2D and sarcopenia, such as poor glucose disposal, insulin resistance, and decreased metabolic rate (21). We therefore included an interaction term (scoreXdiabetes) in the logistic regression together with the main effects. As shown in Supplementary Table S5a, the interaction term was not significant, suggesting that the sarcopenia is independent of diabetes. To confirm this finding, we stratified the population based on diabetes status and found the PAM LoF score significantly associated with sarcopenia only in subjects *without* diabetes [Supplementary Table S5b (13)]; however, the absence of the association in individuals with diabetes could be subject to statistical power issue. Finally, we tested if there was any interaction between PAM LoF score and age (scoreXage), as age itself is the most important risk factor of sarcopenia, finding no significant interaction [Supplementary Table S6 (13)].

## Discussion

The present work represents the largest mapping to date of genetic determinants of PAM-AMA, as measured by a newly developed accurate assay (1). Based on data from almost 8000 individuals from MDC and MPP, we show that carriers of the 2 LoF variants Asp563Gly (5% MAF for MDC-CC and 4% for MPP) and Ser539Trp (0.8% MAF for MDC-CC and 0.6% for MPP) have dramatically reduced PAM-AMA (1.0 and 2.3 SD lower, per effect allele, respectively, estimated in the meta-analysis of the 2 cohorts). Indeed, carriers of these variants (Asp563Gly and Ser539Trp) experience a clinical syndrome characterized by impaired insulin secretion, thereby increasing the risk of developing overt diabetes. This condition is also associated with diminished muscle mass, muscle strength, and sarcopenia and a mildly increased GH and IGF-1 level.

The PAM enzyme consists of 2 catalytic subunits involved in the 2-step amidation reaction: PHM and PAL (1). PHM catalyzes the initial step of the reaction, which involves the hydroxylation of the alpha-carbon in the free C-terminal glycine of a peptidylglycine substrate. Following this, the PAL domain facilitates the second step, which involves the cleavage of the alpha-hydroxylated product, leading to the release of glyoxylate (1). From functional in vitro experiments, the 2 described variants, located in the PAL domain, have been shown to be responsible for a dramatic decrease of PAL activity and for a 2-fold reduction of PHM activity (8). In particular, the Ser539Trp-mutant PAL domain shows 0% activity and Asp563Gly 41% compared to the wild-type (8).

The association between genetic LoF of PAM and diabetes, as well as with glycemic traits (fasting glucose and HbA_1C_ levels), is in line with previous reports (22-24). Moreover, we found that carriers of PAM LoF exhibited a lower insulin response 30 minutes after OGTT. The association with lower early insulin response is concordant with findings from Finnish men reporting that the LoF effect allele of the Asp563Gly was associated with low insulin secretion measured with insulinogenic index [(Ins_30_−Ins_0_)/(Gluc_30_−Gluc_0_)] (25). The finding is also supported by different functional studies emphasizing the role of PAM in beta cell dysfunction and insulin secretion (3), and beta cell lines knocked out for *PAM* showed a reduced ability to mobilize insulin in response to glucose (26).

We also found a suggestive association between an increasing number of PAM LoF alleles and reduced fasting GLP-1 levels, while no association was found with post-OGTT GLP-1. The first result, even if weak, is of some interest since GLP-1 is an amidated hormone, released from intestinal cells, which stimulate insulin secretion in response to meals (3, 27). One can speculate that impaired amidation of GLP-1 may contribute to impaired 30-minute insulin response during an OGTT. We should emphasize that our GLP-1 assay does not distinguish between amidated and nonamidated GLP-1. Our result related to GLP-1 in PAM LoF carriers appears, however, in contrast with what was shown by Umapathysivam and colleagues (unpublished data). In their work, carriers of the 2 PAM mutations had an increased GLP-1 profile after an OGTT and the PAM knock-out mouse exhibited resistance to GLP-1-analogue therapy (unpublished data). Thus, the role of GLP-1 in mediation of genetic PAM deficiency and impaired insulin secretion needs further study.

PAM shows a heterogeneous expression profile across tissues (28). The pituitary gland, the site of production of GH, shows a high expression of PAM (8). Encouraged by the findings of Trivellin and colleagues, who found the Ser539Trp and Asp563Gly substitutions present in sporadic cases with diseases caused by GH excess (ie, acromegaly and gigantism) (8), we assessed the association between PAM LoF and the actual activity of the GH axis at the general population level. We observed an association between PAM LoF and elevated GH and IGF-1 levels, indicating increased activity in the entire GH axis, where GH stimulates IGF-1 production to exert its physiological effects (29). Unexpectedly, PAM LoF carriers exhibited a phenotype more akin to GH deficiency than GH excess. They displayed shorter stature, lower body weight, diminished muscle mass across multiple compartments, and reduced muscle strength (30). This prompts us to speculate that the increased activity of the GH/IGF-1 axis in PAM LoF carriers could be a compensatory secretion mechanism, possibly as a form of positive feedback in response to the loss of muscle mass, rather than a primary consequence of genetic PAM deficiency. An alternative mechanism would be GH resistance, at the receptor level or distally, might explain increased GH and a GH-deficient phenotype.

To gain a better evaluation of the clinical significance of the continuous measures of muscle mass and function, we created a stringent binary trait of sarcopenia and found a strong association with PAM LoF. In particular, the Ser539Trp variant showed a strong association with sarcopenia with a 30% increased risk in carriers of the mutation. Multivariate analysis showed that the effect size was comparable to 2 years of chronological age [Supplementary Table S5b (13)].

Sarcopenia is the age-related progressive loss of muscle mass and function, which commonly coexists with several medical conditions, including diabetes, for which the evidence shows a bidirectional and not completely understood relationship between the 2 factors (31). The most acclaimed theory suggests that either the impaired secretion of insulin in diabetes leads to sarcopenia due to a reduction of the growth-stimulating effect of insulin or that sarcopenic patients might be more prone to diabetes due to the loss of muscle mass that is targeted by insulin action or a combination (32). Importantly, we show that the effect of genetic PAM deficiency is independent of diabetes as the association with sarcopenia was clearly significant in individuals without diabetes. Even though the mechanisms linking genetic PAM deficiency to sarcopenia need further study, the most likely mechanism, based on available data, is the reduced secretion of insulin. In other words, even if genetic PAM deficiency causes sarcopenia in the nondiabetic population, it seems plausible that long-term reduction of insulin secretion is the prime consequence of genetic PAM deficiency, causing an increase in the risk of diabetes and sarcopenia. In support of this, a reduced level of secreted insulin has been reported as a risk factor for developing sarcopenia in Japanese and Chinese older individuals, regardless of their diabetic status (32).

Finally, despite evidence from animal studies showing that cardiomyocyte knockout of the PAM gene resulted in impaired secretion of natriuretic peptides (4), we found no association between genetic PAM deficiency and circulating levels of the natriuretic peptides MR-ANP and Nt-BNP. More studies are needed to map any long-term cardiovascular consequences from genetic PAM deficiency, not the least since diabetes is a well-known risk factor for cardiovascular disease (33), and elevation of GH at the population level, which we observe here as a possible compensatory effect of PAM deficiency, has been shown to predict cardiovascular morbidity and mortality (17, 34).

Our study has several limitations. First, not all phenotypes were measured in every cohort, which poses a constraint on the comprehensiveness of our analysis. Additionally, the generalizability of our findings to non-European populations is restricted, as our investigation only encompassed European cohorts. Lastly, with the existing data, we are unable to discern whether the LoF mutations directly impact enzymatic activity or indirectly influence enzyme concentration. Nevertheless, this is the largest study performed thus far to reliably identify genetic determinants of circulating PAM-AMA deficiency, offering precise effect estimates for 2 mutations. Apart from verifying previously reported associations with a higher risk of insulin-deficient diabetes mellitus, we also expand our understanding of the clinical consequences of genetic PAM deficiency in sarcopenia, a growing medical problem and a major cause of disability, hospitalization, and healthcare costs (26, 35). Aging, and its consequences, is by far the most important recognized cause of sarcopenia, and genetic PAM deficiency would only explain a negligible proportion of all sarcopenic cases in society (35). Still, our results point to one of the first specific molecular aetiologies behind sarcopenia that is genetic in nature and can be traced even in the early stages of life. This calls for more research on possible ways to modulate (increase) PAM activity as a possible preventive therapy for sarcopenia. Moreover, given that physical activity is widely regarded as the most effective prevention strategy for sarcopenia and is also a modifiable risk factor for diabetes (35-37), it is reasonable to speculate that early-in-life targeted exercise interventions aimed at prevention of both sarcopenia and diabetes would be an interesting precision prevention approach to scientifically test in individuals with genetic PAM deficiency, such as those 0.7% of all Europeans who carry the Ser539Trp mutation and have a 30% increased risk of sarcopenia.

In conclusion, genetic PAM-AMA deficiency results in a prediabetic sarcopenic phenotype. Early identification of carriers of such mutations would allow tailored exercise interventions and calls for novel therapies that restore PAM-AMA activity in PAM LoF mutation carriers. Moreover, our findings encourage research on pharmacological approaches to enhance PAM activity to mitigate the increased risk of diabetes and sarcopenia at least in individuals carrying such mutations.

## Data Availability

Some or all datasets generated during and/or analyzed during the current study are not publicly available but are available from the corresponding author on reasonable request.
